# Phase correction in violin ensemble performance

**DOI:** 10.1038/s41598-026-46205-6

**Published:** 2026-05-04

**Authors:** M. S. Li, D. Ward, R. Stables, W. L. Chua, J. Howe, K. A. Quinn, A. M. Wing

**Affiliations:** 1 Sensory Motor Neuroscience Lab, School of Psychology, University of Birmingham, Birmingham, B15 2TT UK; 2 School of Computing and Digital Technology, Birmingham City University, Birmingham, UK; 3 Department of Psychology, DePaul University, Chicago, USA

**Keywords:** Timing, Rhythm, Ensemble, Synchronisation, Joint action, Human behaviour, Perception

## Abstract

In the absence of a conductor, members of a music ensemble need to take their timing cues from each other in order to play together. These timing cues may be visual or auditory. This paper investigates the use of auditory cues in the absence of visual cues. We applied a Linear Phase Correction Model^1^ to investigate the effects of performance mode and rhythmic complexity on how violin-playing participants synchronise to a violin duo. Participants played the melody part of a 48-note musical excerpt in synchrony with a violin duo playing melody and accompaniment, in which we manipulated the metrical structure to form a simple or complex rhythmic relation between melody and accompaniment player. We also varied the performance mode of the melody player (live vs. recorded) while the accompaniment player was always recorded. After each trial, participants rated the perceived influence of the melody player and the accompaniment player in the duo. Participants exhibited lower correction gain to the accompaniment player in the complex rhythmic condition. This effect was more pronounced when the melody player was live. Complementing this finding, ratings indicated greater perceived influence of the live melody player than the accompaniment, especially in the simple rhythmic condition. These patterns of correction and perceived influence indicate that players favour taking correction cues from the score part that is more similar to their own in terms of melody and rhythmic structure.

**Supplementary Information:**

The online version contains supplementary material available at 10.1038/s41598-026-46205-6.

## Introduction

Research in music cognition has established the importance of simple timing models for understanding how performers achieve synchrony, with seminal contributions from Palmer^2^, Schulze et al^3^, Repp^4^, Keller^5^ laying the groundwork for contemporary investigations. Simple sensorimotor synchronization tasks^6^–^8^, such as tapping along to auditory rhythms, have been central to this work, revealing that musicians generally exhibit greater timing accuracy, more stable entrainment, and more efficient phase correction mechanisms than non-musicians, reflecting well documented links between perceptual and motor processes in rhythmic coordination^9^. We consider a simplified timing model paired with a tightly constrained music ensemble performance task as a link to studying more naturalistic live music performance.

Recent research shows that microtiming and synchrony in music performance emerge from a complex interplay of sonic, perceptual, and contextual variables that are difficult to disentangle in naturalistic settings. For instance, Danielson and colleagues^10^ demonstrate that factors such as attack shape, timbre, and sound morphology systematically shift perceived beat placement, revealing how many interacting dimensions must be controlled before meaningful inferences about performer coordination can be drawn. This suggests potential for an approach based on a simplified model that isolates core timing mechanisms before attempting to interpret the richer, layered timing behaviors found in real ensemble performance (e.g. Danielson and colleagues^11^). By starting with a constrained paradigm, researchers can build rigorously validated foundations that later support more ecological, complex studies, ultimately bridging controlled experimentation with the dynamic synchrony observed in live small group performance.

Interpersonal synchronisation is an act of aligning one’s actions with other interacting persons in time^12^, which is a common characteristic of many social interactions. Small ensemble performance of western classical music, such as string quartet (2 violins, viola and cello) chamber music, is a prime example of such social interaction^13^. Each player adjusts their timing to coordinate with their partners and achieve joint expressive goals without external indication of a common timing metric (i.e. conductor). Musical scores are usually notated with standard tempi, such as *allegro* for very fast and lively performances and *andante* for moderately slow music. However, actual tempi used during performances may depart from the score indication. For instance, players might adjust their own tempo intentionally to the dynamics in music (e.g. loudness, pitch) and to show their own interpretation of musical expressiveness (e.g. tempo changes marking the beginnings and ends of phrases). In this paper, we study how ensemble players coordinate in time to maintain group cohesion despite such individual variation in timing.

The quality of interpersonal synchronisation is commonly evaluated by the *mean* and *variability* of asynchrony values between players. In wind and string trios, for example, Rasch^14^ showed that note onset timings varied between players with a mean (signed) temporal asynchrony of up to 20 ms, and a standard deviation of asynchrony up to 50 ms. In comparison to note durations running into hundreds of milliseconds, mean asynchrony is relatively small and allows the ensemble to reflect the role of leadership or musical expression^15^. Despite expressiveness being carefully practiced and controlled, attention deployment and technical challenges during live performances can introduce fluctuations in timing. We assume ensemble players aim to minimise their variances of asynchrony with other players, although we accept that some deviations from this principle may occur at points of expressive interpretation. In this study we investigate how the relative phase of the note onsets of a participant playing melody on the violin, is maintained with respect to two other violinists, one also playing the melody and the other an accompaniment, whose rhythmic relation to the melody was manipulated.

Cues to synchrony in ensemble performance which players might use to adjust their timing may be visual or auditory. A strategy sometimes employed with coaching chamber quartets is to turn the chairs on which the players are sitting so they are back to back, removing visual timing cues^16,17^. This practice is considered to improve listening skills which are recognised to be very important in chamber music^18,19^. In the present paper we removed visual cues from participant players by placing them in a cubicle where they had no visual link with two other players in the study. This allowed us to focus the analysis on the auditory dimension of timing regulation unconfounded by visual factors.

**Fig. 1:**
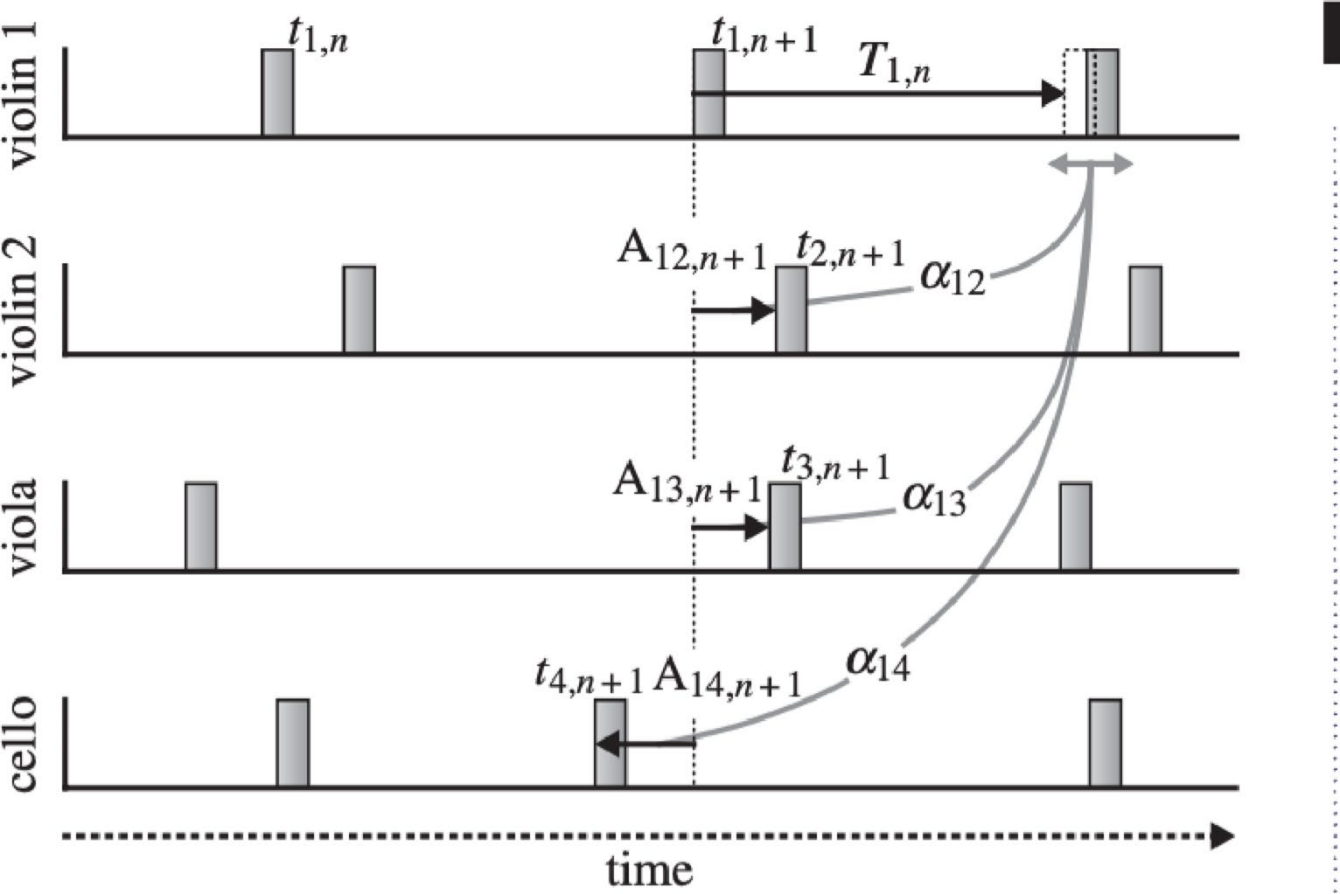
Diagram of the phase correction model (Wing et al^1^.) for quartet synchronization, shown from the viewpoint of violin 1. The four panels in Y correspond to the four players in a quartet show successive notes (without regard to beats or divisions) to be played in unison, and the X-axis indicates time with asynchronies relative to violin 1 note at time t_1,*n*+1_ represented by A_12,*n*+1_ .etc.

Wing et al.^1^ modelled the process of ensemble synchronisation in string quartets with a first-order Linear Phase Correction Model. See Eq. (1) below for the original notation:

1 $${t}_{i,n}. ={t}_{i,n-1}+{T}_{i,n}-\sum_{j=1,j\ne i}^{4}{\alpha\:}_{ij}\left({t}_{i,n-1}-{t}_{j,n-1}\right)+{\epsilon\:}_{i,n},\ i =1...4$$

where $\:{t}_{i,n}$​ and $\:{t}_{i,n-1}$​ are current and previous note onset timing for player *\:i*, $\:{T}_{i,n}\:$represents the timekeeper interval, $\:{\alpha\:}_{ij}\:$refers to the correction gain applied by player *\:i* for the asynchrony $\:{t}_{i,n-1}-{t}_{j,n-1}$with player$\:\:j$, and $\:{\epsilon\:}_{i,n}$​ is a random noise term identified with the assumed internal timekeeper. Figure 1 shows the schematic of the phase correction model for four players’ synchronisation from the perspective of player one (violin 1), taken from Wing et al.^1^. This model assumes each player adjusts the timing of their next note onset in weighted proportion to their asynchronies on the previous note onset with each other player. In this model, optimal correction, which minimises asynchrony variance, is achieved with correction gain given by one divided by the total number of players (four). Thus, in the case of quartets studied by Wing et al., the optimal value for the gain was 0.25.

Two professional quartets performed a short musical excerpt (48 notes with homophonic scoring from Haydn Op 74 No.1 fourth movement) with unrehearsed expressive variation over a series of 15 repetitions. Results showed that players’ temporal corrections were broadly optimal while performing; that is the average values of the correction gains used by each of the two quartets were close to 0.25. The estimated gains of the individual players helped to identify the coordination strategy of a particular ensemble. For instance, Wing et al.^1^ reported one of the two quartets exhibited relatively stronger timing adjustments to the leader whereas the gains were more balanced amongst the players in the other quartet. This was taken to imply that ensemble synchrony may be achieved, sometimes with a more hierarchical organisation (as in the first quartet), and at other times with a more cooperative approach (as in the second quartet).

Correction gain values are not necessarily fixed or equal between players. In the present study, we were interested to identify the factors that influence the level of gain used by a player in an ensemble. We investigated this by asking a participant player, who was assigned to play the melody part, to join with a pre-formed duo comprising a melody player and an accompaniment player (i.e. the participant’s part was in unison with the upper part of the violin duo). There were three factors: player (melody vs. accompaniment), rhythmic complexity (simple vs. complex) and mode (live vs. pre-recorded playing) of the duo. Taking these in turn, first, when the participant player is playing in unison with the melody part of the duo, we expected that the participant player would be likely to find the melody player of the duo more salient than the accompaniment player. We thus expected the participant to apply higher correction gain to the melody player compared to the accompaniment player. Second, in terms of the rhythm, we asked what if the accompaniment player contrasts with the participant by playing in cross-rhythm? We expected that the correction gain used by the participant relative to the accompaniment would be weakened if the accompaniment had a cross-rhythm with that of the melody.

The third factor, mode, explored the direction of information flow for timing correction between players. In Wing et al.^1^, each player was free to correct to each of the other players and they, in turn, to correct to that player; but what if one or more of the other players do not correct? For instance, in the case of piano duets Goebl and Palmer^20^ reported increased asynchrony variability with reduced auditory timing feedback from another player. Here, we examined whether correction gain was also affected by limited information flow, in which we manipulated the melody player in the duo who was either playing live (and adapting to the participant) or was pre-recorded. We expected that, when the melody was recorded (and so not adapting to the participant), the participant might increase their correction gain to maintain the overall stability of the group.

We were interested in the participants’ subjective experience of the different ensemble conditions. Players were asked after each trial to indicate whether they thought the melody part was live or recorded. We expected that correction gain might be higher if participants were able to correctly identify the melody as recorded, knowing that their partner was not adapting to them. We were also interested in the degree to which participants were sensitive to the corrections they were making to the other two players. After each trial we also asked them to rate the degree to which they experienced the melody and the accompaniment as influencing their performance.

In summary, this study is concerned with ensemble timing measures of the effects on synchronisation behaviour of rhythmic complexity and live vs. recorded partners based on auditory cues in the absence of visual cues. The measures include (1) participant-melody and participant-accompaniment temporal asynchrony of note onsets, (2) correction gain values of the participant player with respect to the melody and the accompaniment player, estimated by the linear phase correction model, and (3) ratings of the subjective experience of ensemble.

## Methods

### Participants

A total of 9 amateur musicians (5 self-reported males, 4 self-reported females, age-range 18–65 years) participated in the study and were rewarded with a payment of 7 pounds per hour. All participants were naïve to the purpose of the study and self-reported as grade five (or above) violinists in the Associated Board of the Royal Schools of Music graded examinations (see https://www.abrsm.org/en-gb/practical-grades/about-practical-grades for further information about practical grades). Informed consent was obtained before the start of the experiment. The experiments were conducted according to the protocol approved by the Science, Technology, Engineering and Mathematics (STEM) Ethical Review Committee of the University of Birmingham and the Declaration of Helsinki.

### Apparatus

All audio samples in this study, including the pre-recorded samples and live performances, were acquired with the same set-up. Audio signals, sampled at 48 kHz with a bit depth of 16 bits and stored in WAV file format, were presented through Samson SR950 headphones. DPA 4099 condenser microphones were mounted on the body of each violin close to the f-hole opposite to the bow arm and connected to an Allen and Heath Zed24 audio mixer interfaced with an iMac computer running Logic Pro audio production software.

Following Wing et al.^1^, a 48-note excerpt from the Op. 74 No. 1 quartet of Joseph Haydn (fourth movement, bars 13-24) was employed with the first violin part used for the melody player (and the participant player) and the second violin part for the accompaniment player (see Fig. 3A). The 48-note excerpt included the pickup note in bar 12 (note C for the melody and participant player, note E for the accompaniment) and the first 3 notes in bar 24. To keep temporal alignment across parts, the second and fourth demisemiquaver notes in bar 23 of the original first violin score were removed, resulting in all parts containing a total of 48 notes. The original excerpts were scored as homophonic which is helpful for the study of synchronisation, and the standard tempo of all performances in this experiment was set to a relatively leisurely 100 bpm.

### Set-up

During the experiment, the performances of the melody player were presented to the participant’s left ear (for both recorded and live tracks), and the accompaniment player’s recordings to the right ear. The melody player, when performing live, was also presented with the participant player’s performances and the accompaniment player’s recordings through headphones. The live audio from the melody player was sent directly to the participant’s headphones through an analog mixing console to prevent any computer-induced digital delay.

### Experimental design

**Fig. 2:**
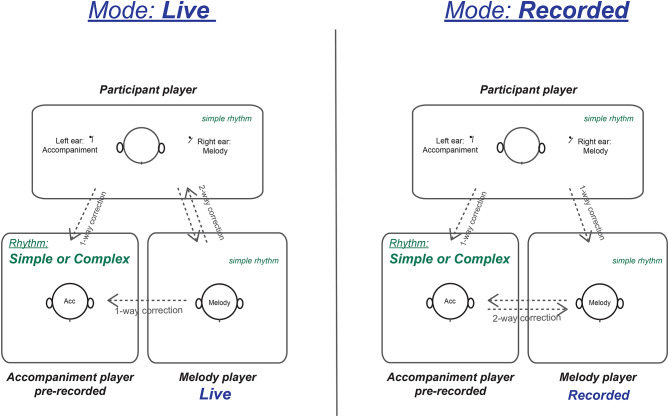
Experimental design. Links between the participant player and the duo, in which the performance mode of the melody player is either live (left) or recorded (right) and the rhythmic relation between melody and accompaniment player is either simple or complex. Dashed lines suggest possible correction terms between players where the arrowhead indicates correction target.

The participant player (Fig. 2 top rectangle), who was assigned the melody line of the duo’s score, joined the performance of an unseen violin duo, in which there was a melody player who played the first violin’s melody part (i.e. the participant played in unison with the melody player), and an accompaniment player who played the second violin accompaniment part (Fig. 2 bottom squares). The performance mode of the melody player (within-participant, 2 levels) and the rhythm of the accompaniment player (within-participant, 2 levels) were manipulated as detailed below.

**Fig. 3:**
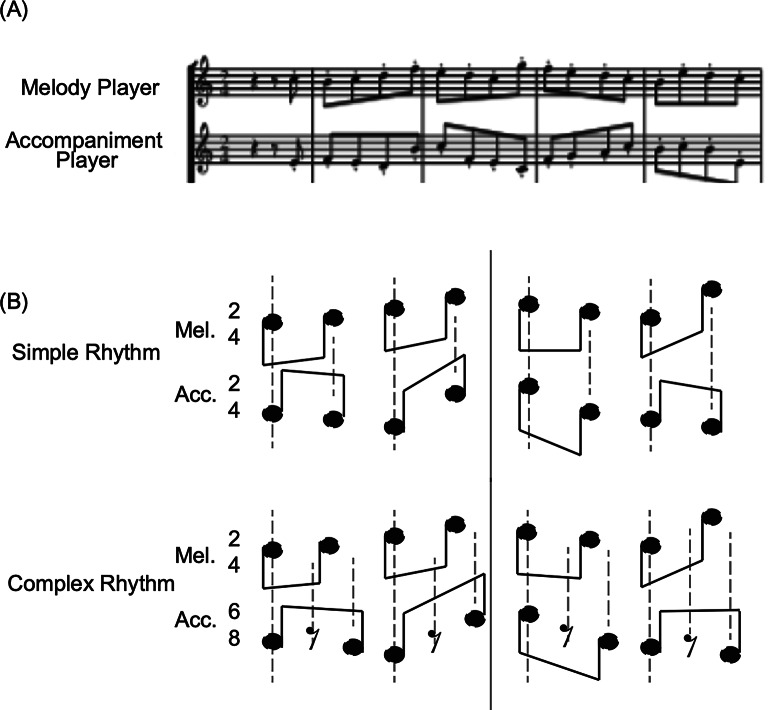
Methods. (**A**) Beginning 4-bar section of the 48 notes excerpt from Joseph Haydn No. 74 No. 1 quartet fourth movement (bars 13–24 with pick-up note from bar 12), in which the first violin part (top stave) was assigned to the melody player, and the second violin part (bottom stave) to the accompaniment player of the duo. (**B**) Two rhythm conditions, where both parts perform in 2/4 time signature for the simple condition (top panel), and the accompaniment part was revised to be a 6/8 time signature for the complex condition (bottom panel).

The excerpts were written in either simple time or compound meter for the accompaniment player, which resulted in either a simple or a complex rhythmic relation between melody and accompaniment player. For the simple condition, the melody and the accompaniment player both played in 2/4 time (i.e. four quavers in a bar, each equally spaced with an Inter-Onset Interval (IOI) of approximately 300ms, on the basis of the metronome at the beginning of the trial). For the complex condition, the melody part was scored in 2/4 time, and the accompaniment part was revised and scored in 6/8 time. That is, a crossrhythm was created by the accompaniment with six quavers in a bar equally spaced with an IOI of approx. 200ms, where the four notes in a bar were placed on the first, third, fourth and sixth quavers, with quaver rests replacing the second and fifth quaver notes as shown in Fig. 3B. The participant player’s melody score was written in 2/4 time, in the same way as it was for the melody player.

The performance mode of the melody player could be either a recording (Fig. 2 right panel) or live performance (Fig. 2 left panel). In other words, while the accompaniment player was always a pre-recorded track, the melody heard by the participant was either pre-recorded or live. In both cases, the sounds were presented to the participant player through headphones. For the pre-recorded conditions, 10 takes of each of the simple and the complex conditions were recorded by the duo performing together. For the (melody) live conditions, another 10 takes were acquired for each of the simple and complex conditions with the accompaniment player alone. Ten auditory samples were required to ensure that participants would not adapt to a single recorded track over repetitions. By testing with a range of samples participants would have to make active efforts to achieve synchronization at each take, rather than possibly learning a single recording, which could have made synchronizing to the same sample progressively easier over time. Across all repetitions, the duo were instructed to play metronomically, without expressive phrasing, and staccato (i.e. short and detached) to aid onset detection.

### Procedure

The performers of the duo were provided with their respective parts in advance of the recording session. Both the melody and accompaniment players in the duo held ABRSM Grade 8 qualifications and received the musical score two weeks prior to the recording day, allowing sufficient time for individual practice. The duo did not practice together prior to the recording. The duo recorded the 48-notes excerpt with an eight quaver beat lead-in from a metronome in which they listened to the first four, and started playing the note C repeated with the fifth through eighth, followed by the excerpt. The accompaniment player was recorded on their own for the samples where the melody player would perform live. The duo were recorded together for the samples where both the melody and the accompaniment were pre-recorded. Throughout the recording session, the duo were instructed to play staccato, and use steady timing without expressive phrasing.

Participant players were also provided with music score for their part (first violin) two weeks before the study for familiarization purposes. No instructions were given about musical expressiveness or the tempo of the excerpt. On arrival the participant was informed that there was another violinist who, in playing the melody, would sometimes perform live, sometimes as a recording. The melody performer and the participant played in separate recording cubicles throughout the study to avoid any visual and verbal contacts. As a warm-up exercise, participants were asked to play single staccato notes on open D-string to a 100-bpm metronome for five minutes before the experiment. When playing with the duo, participants were asked to play staccato (short, detached notes) and to keep in time with the other two players.

Each trial began with eight quaver beats from a metronome. Participants were instructed to listen to the first four beats and then join in from the fifth beat, playing four note C four times before proceeding to the Haydn excerpt (48 notes in total; see Fig. 3A). The participant player was accompanied by the violin duo over the headphones. The two variables, mode and rhythm, were manipulated in a 2 × 2 within-subjects design, where the four conditions (live/simple; live/complex; recorded/simple; recorded/complex) were randomly repeated with counterbalanced order ten times, resulting in 40 trials per participant. At the end of each trial, participants were asked to indicate whether they felt that the melody player was a live performance or a recording on that trial. Participants also had to provide ratings for the degree to which each of the melody and accompaniment players influenced their playing on that trial. A Likert scale was used in which the two extreme ends (− 3,+ 3) were labelled ‘negative distraction’ and ‘positive influence’ and middle of the scale (0) was to be considered neutral in terms of influence.

### Onset detection

A two-stage process was used to transform the raw audio data into a series of time points corresponding to the onset of each note. The first stage used the high-frequency content spectral method^21^ for onset detection as an initial estimate of the onset times for a sample of trials from multiple players. False positives were removed manually by an experienced sound engineer using Sonic Visualiser^22^. Second, a time-domain procedure, with parameters optimised using the results from the first stage, was used to estimate the true onset times. In this procedure, mid-frequency resonance was reduced by applying a third-order bidirectional Butterworth high-pass filter with a cut-off frequency of 3 kHz. This was done to better capture distinct attacks in the amplitude envelope, which were otherwise smoothed out by the resonance response of the violin body and therefore making onsets less easy to detect. An amplitude envelope was then obtained by applying a bidirectional exponential-moving-average filter with a time-constant of 3 ms to the full-wave rectified, high-pass-filtered audio. The logarithm of the envelope was taken, and subsequently down-sampled using a time-step of 1ms to obtain the onset-detection function^23^. Median filtering was performed to select peaks from the onset-detection function, yielding onset times of the player’s notes. Detection parameters such as smoothing constants and median-filter thresholds were optimised using the onset data obtained from the first stage as ground truth. Finally, three audio technicians inspected and verified the dataset by comparing each onset time-series (for every recording) with the corresponding temporal waveforms and spectrograms.

### Timing analysis

In each trial 52 notes were recorded, where the first four notes (i.e. quaver beats of note C) were discarded from the analysis. The note onsets were derived with the methods described above. To ensure that the timing structure was consistent across all players and conditions for statistical analysis, the accompaniment note onsets in the 6/8 time signature were transformed to the timing structure of the melody player (in 2/4 time signature) via linear interpolation (see Zamm et al.^24^ for a similar interpolation approach for analysing half-beat notes). The linear interpolation approach estimates the onset time of the second quarter note in a bar by a weighted average of the two surrounding notes, which allowed us to use all available data for timing analysis, rather than discarding portions of it.

The performance timing of each participant was studied by deriving 47 IOIs from the difference between 48 note onsets for each trial. Following Zamm et al.^24^, IOIs exceeding three standard deviations of the mean within a condition were treated as performance errors and removed (the mean error rate was 0.25%). We also calculated asynchronies between players by subtracting the melody (or accompaniment) note onsets from the participant’s onsets, in which a negative asynchrony indicated that the participant was playing *ahead* of the duo. The IOI values reflected how performance tempo varied across experimental conditions, whereas the asynchrony values reflected how the participant-duo relationships were challenged by rhythm complexity and performance modes.

Statistical results were summarised with 3-way repeated measures ANOVA with mode, rhythm, and player as factors and using *p*=0.05 as significance level. Follow-up t-tests were used to determine significance of mean differences between conditions.

### Linear phase correction model

The Linear Phase Correction Model (Eq. 1) was rewritten in alternative form by Jacoby et al.^25^ that allows estimates of timekeeper and motor variance assuming that motor variance is almost always smaller than timekeeper variance:

2 $$\:{R}_{k+1}=\sum\:_{i=1}^{P}\:-{\alpha\:}_{i}{A}_{k}^{i}+{H}_{k}$$

where

$$\:{H}_{k}={T}_{k}+{M}_{k+1}-{M}_{k};$$

$$\:{M}_{k}\sim\:N(0,{\sigma\:}_{M}^{2});{\:T}_{k}\sim\:N(0,{\sigma\:}_{T}^{2})$$.

This was applied to the empirical note onset timings (*R*) to measure the degree of timing adjustment between participant-melody player and participant-accompaniment player (with number of players *P*). Here, in contrast to Wing et al.^1^ who used an iterative least squares procedure for parameter estimation, a bounded General Least Squares (bGLS) method was implemented to avoid estimation error and bias (see Jacoby et al.^25^ for a comparison between the two estimation methods). Estimates of two correction gain parameters ($\:\alpha\:$) were obtained from each trial (one correction term for each of the players in the duo), assuming the asynchrony (*A*) between the participant player and the duo (*\:i*) of current interval *k + 1*, was adjusted by the asynchronies and correction gains of all other players in the previous interval *k*. Mean asynchronies were computed separately for each condition (and for each participant) by applying bGLS to the empirical data.

With the bGLS estimation method, it was also possible to obtain an estimate of timekeeper ($\:{\sigma\:}_{T}^{2}$) and motor variance ($\:{\sigma\:}_{M}^{2}$) values to understand the contribution of the internal generation of time intervals and motor execution of successive responses to timing variance. As shown in Supplementary Fig. 1A, the results indicated that motor variance was approximately half that of the timekeeper, with motor variance ranging from 20 to 100 ms^2^, in line with previous findings obtained with tapping and ensemble synchronisation tasks^26–30^. Our results also showed that the motor variance remained relatively constant across the four conditions in comparison to timekeeper variances. In this paper the focus is on correction gains, and we do not analyse motor and timer variances further.

The estimated correction gain values were plugged into Jacoby et al.^25^ multi-person linear phase correction model (Eq. 2) to reconstruct the model’s prediction for IOIs. The goodness-of-fit between empirical IOI values observed for each participant and the predicted IOI values was then determined by computing the R-squared values as a measure of how much variance of the empirical data could be accounted for by the model. The average R^2^ values across participants were 0.301 (SD 0.06) for live/simple, 0.271 (SD 0.05) for live/complex, 0.287 (SD 0.06) for recording/simple, and 0.265 (SD 0.04) for recording/complex.

## Results

We first summarise timing in the *duo performances* as reference stimuli. We then focus on the participants’ timing results.

### Reference stimuli: performance of the duo

The mean IOI values of the melody player of the duo averaged over live samples were 288.3 ms (SD 1.8 ms) for the simple, and 302.2 ms (SD 3.4 ms) for the complex condition. For the recorded samples, they were 282.1 ms (SD 2.9 ms) for the simple, and 294.5 ms (SD 2.0 ms) for the complex condition.

**Fig. 4:**
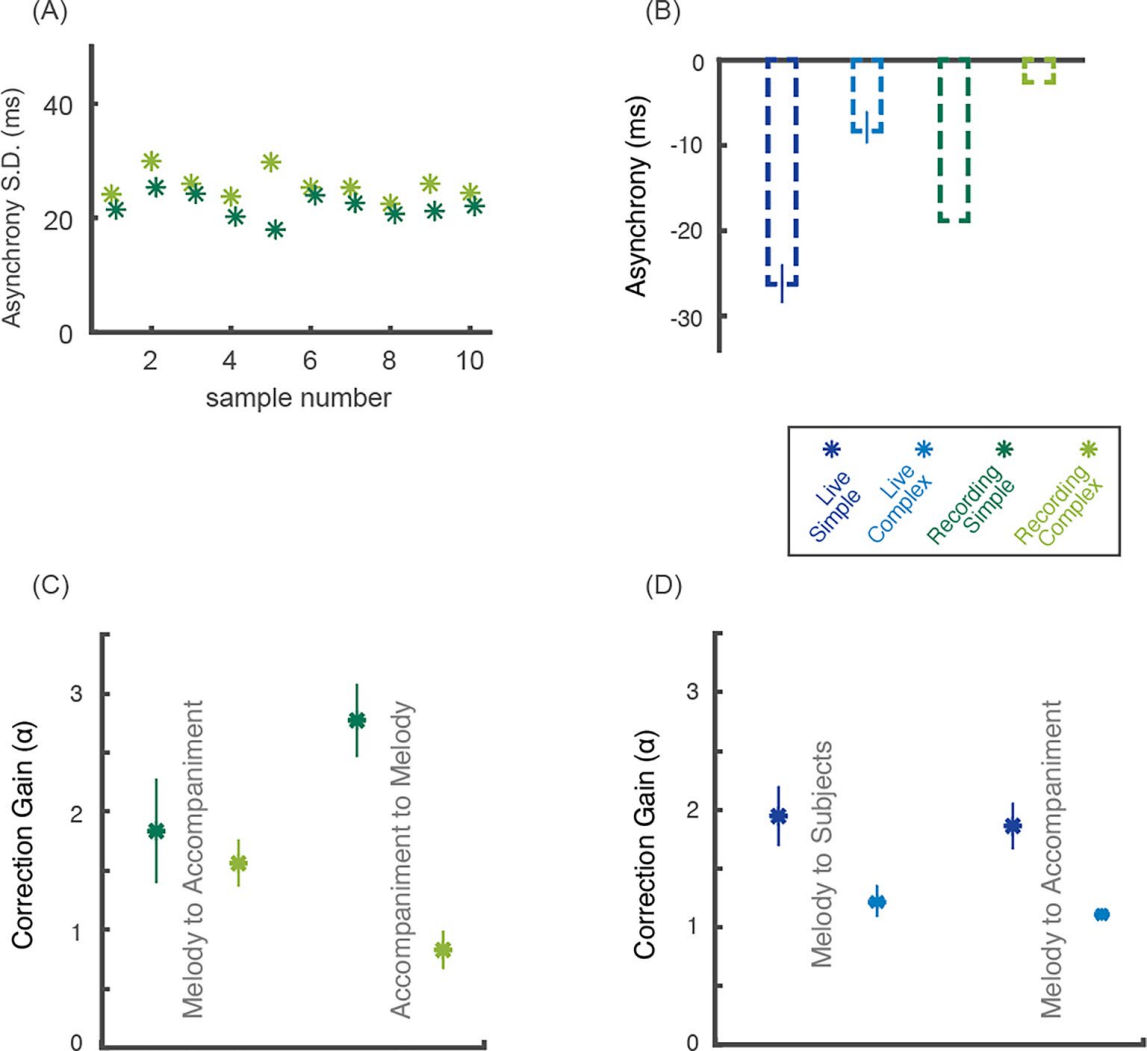
Summary of duo performance. (**A**) Asynchrony variability (x-axis) in a trial as a function of recording sample number (y-axis) for the simple (in dark green) and the complex (in light green) condition. (**B**) Asynchrony between the melody and the accompaniment player, where a negative value indicates the melody leads. Error bars are ± 1 SE across takes. (**C**) Correction gain parameter of the duo during the recordings, where error bars are ± 1 SE across samples. (**D**) Correction gain of the live melody player, where error bars are ± 1 SE across repetitions.

Duo recordings exhibited stability in asynchrony variability over the 10 repetitions (see Fig. 4A, where asynchrony SD on the y-axis remained relatively consistent and without any overall trend across the 10 samples as plotted on the x-axis). Figure 4B shows that the melody player led the accompaniment player in all conditions as shown by the *negative* mean asynchrony: in live/simple by 26.2 ms, live/complex by 7.9 ms, and recording/simple by 18.7 ms, recording/complex by 2.6 ms.

For the recording samples, the melody player’s correction gain for the accompaniment and the correction gain of the accompaniment player to the melody player can be seen in Fig. 4C. During the experiment, the live melody player’s correction gain to participants and to the accompaniment recordings are shown in Fig. 4D.

### Participant performance

#### IOIs

Participant’s performance timings generally reflected the tempo of the duo (i.e. faster in the simple, and slower in the complex condition): live/simple 288.5ms (SD1.3ms), live/complex 302.2ms (SD 5.5ms), recording/simple 281.6ms (SD 3.0ms), recording/complex 294.9ms (SD1.9ms).

**Fig. 5:**
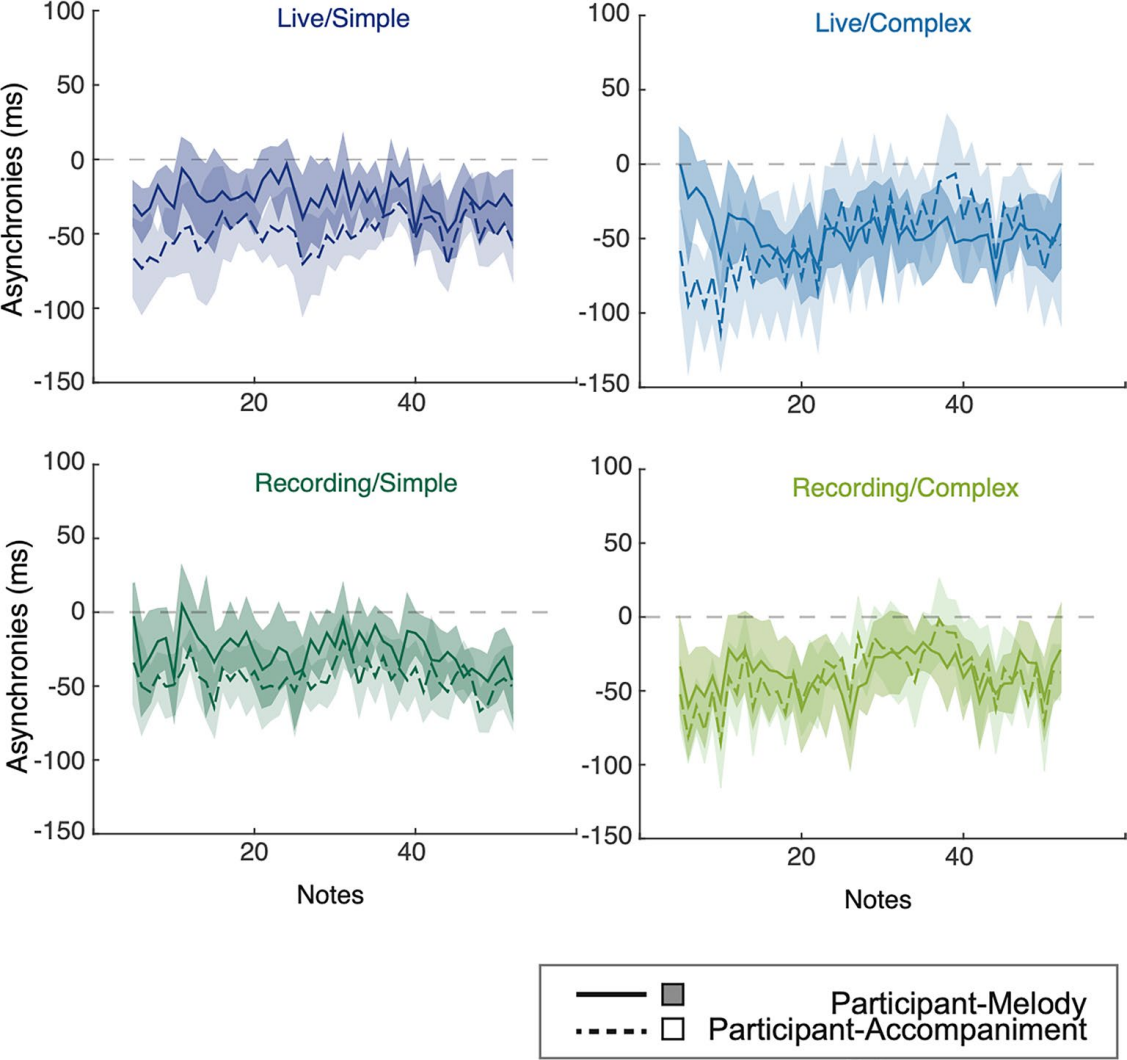
Mean asynchrony. Time series for one participant as a function of notes in a trial, where the note numbers are shown on the x-axis and asynchronies (in ms) are on the y-axis. Thin lines are the mean and the shaded area associated with it are 1 SD across repetitions. The asynchronies between participant and melody are shown with filled lines (with darker shaded areas for SD), and the asynchronies between participant and the accompaniment are shown in dashed lines (with lighter shaded areas for SD).

#### Asynchronies

**Fig. 6:**
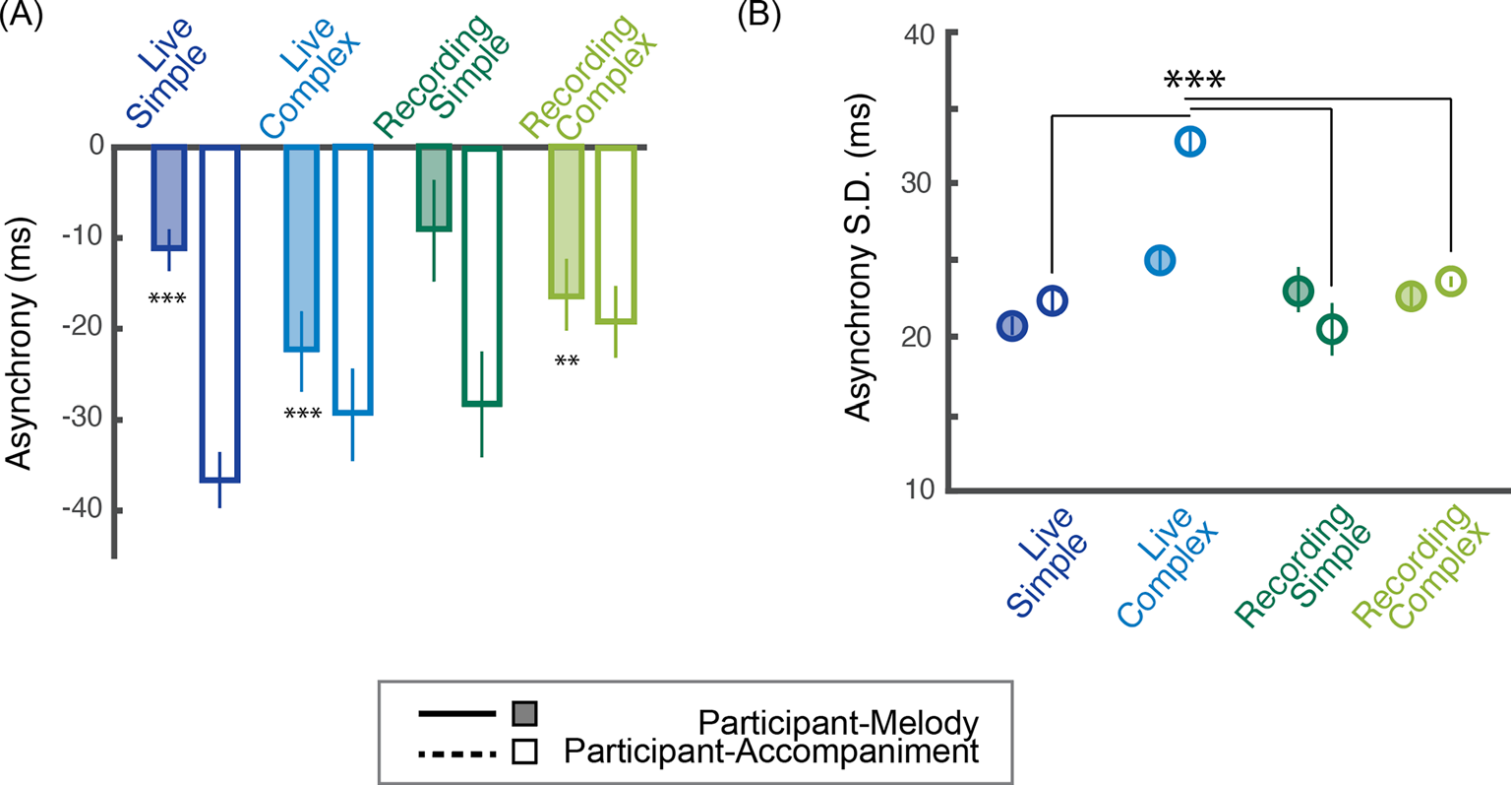
Asynchrony and asynchrony variability. (**A**) Mean asynchrony across participants between the participant player and the duo, where negative asynchrony indicates participant-lead. Error bars are 1 SE across participants. Asterisks indicate statistically significant differences from 0. All four conditions are presented in different colours, from dark blue (live/simple), light blue (live/complex), dark green (recording simple) and light green (recording complex). (**B**) Mean asynchrony variability in each trial averaged across repetitions of all participants, error bars are 1 SE. Participant-Melody asynchrony SD are illustrated with filled circles, and participant-accompaniment asynchrony SD are illustrated with empty circles. The asterisk indicates statistically-significant differences using paired-sample t-tests.

To give a general sense of participant performance, and how asynchrony fluctuates across repetitions, illustrative single trial time series of the asynchronies of one participant are shown in Fig. 5, where it can be seen that, on average, the negative asynchrony (Y-axis) is ~ 30 ms and tends to be more negative (greater lead) with respect to the accompaniment (dashed lines with lighter shaded areas). In addition to the consistent lead of the melody over the accompaniment player, results also showed that participants mostly played ahead of the melody player in all conditions except the recording/simple condition, as shown in Fig. 6A.

#### Asynchrony variability

A three-way repeated measure ANOVA indicated that the effects of performance mode (*F*(1,8) = 9.0, *p* = 0.017), rhythm (*F*(1,8) = 19.6, *p* = 0.002), and player (*F*(1,8) = 18.3, *p* = 0.003) on asynchrony SD were statistically significant. The interactions between mode and rhythm (*F*(1,8) = 14.0, *p* = 0.006), mode and player (*F*(1,8) = 130.0, *p* = 0.000), and rhythm and player (*F*(1,8) = 54.7, *p* < 0.001) were also significant. The interaction between the three factors was not significant (*F*(1,8) = 1.4, *p* = 0.156). Figure 6B shows the asynchrony variability of participant-melody (filled circles) and participant-accompaniment (empty circles) of all four conditions tested. It shows that, generally, participant-accompaniment asynchronies are more variable than participant-melody (i.e. empty circles are higher than filled circles in most conditions), and that participant-accompaniment asynchronies are the most variable in the live/complex condition (lighter blue circles in Fig. 6B).

#### Linear phase correction model correction gain

**Fig. 7:**
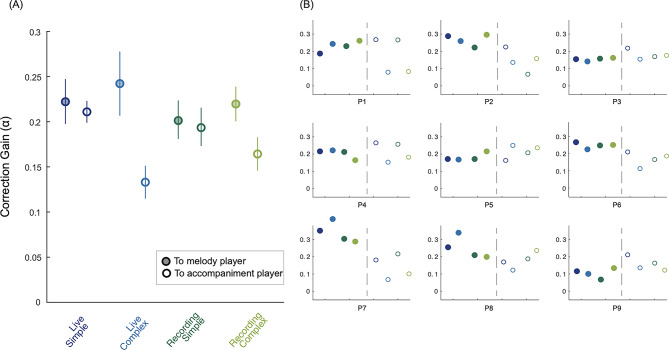
Estimated correction gains. (**A**) Mean correction gain of participant player to the melody player (in filled dots) and to the accompaniment player (in empty dots). Error bars are 1 SE across participants. (**B**) Individual correction gains to the melody player (filled dots; left sub-panels) and to the accompaniment player (empty dots; right sub-panels); in each panel the order of conditions is live simple, live complex, recorded simple, recorded complex.

The overall average correction gain was 0.20. Fig. 7A shows the mean correction gain observed in each of the eight conditions. Two-way repeated measure ANOVA indicated an interaction between player and rhythm (*F*(1,8) = 6.6, *p* = 0.033). No significant main effects or other interaction terms were found; performance mode (*F*(1,8) = 1.4, *p* = 0.274), rhythm (*F*(1,8) = 1.7, *p* = 0.226), player (*F*(1,8) = 2.8, *p* = 0.134), interaction between players and modes (*F*(1,8) = 0.9, *p* = 0.361) and between modes and rhythms (*F*(1,8) = 1.7, *p* = 0.235). We also found no significant interaction between three factors (*F*(1,8) = 3.6, *p* = 0.095).

The source of the reliable two-way player and rhythm interaction was identified using paired-sample Bonferroni corrected t-tests. This showed no significant difference between melody-accompaniment in the simple rhythmic conditions (live/complex vs. live/simple t(8) = − 1.1, *p* = 0.283), but a reliable difference between melody-accompaniment in the complex rhythmic conditions (live/complex vs. live/simple condition t(8) = 3.1, *p* = 0.015). This effect can be observed in Fig. 7A, in which the correction gain to the accompaniment player (empty circles) decreases from the live/complex condition (dark blue) to the live/simple condition (lighter blue). The results suggest that participant players’ correction gains to the accompaniment player varied according to simple or complex conditions, but their correction gains to the melody player were less affected by the rhythmic factor.

The individual results in Fig. 7B show that the participant to melody correction gain (left sub-panels) were relatively consistent across the four conditions for most individuals. The right sub-panels show that, for the live condition, 8 of 9 participants employed a lower correction gain to the accompaniment in the complex condition compared to the simple condition. In the recorded condition, 4 of 9 participants also showed lower gain in the complex compared to the simple condition.

A negative correlation was observed between the participants’ gains with respect to the melody and the accompaniment. This was consistently more negative (p=0.033) in the complex condition (*R* = − 0.47) compared to the simple condition (*R* = − 0.26).

### Subjective perception

#### Live vs. recording discrimination

Overall, participants correctly discriminated whether the melody was live or recorded on 60.5% of trials. A two-way repeated measure ANOVA showed that discrimination of live and recorded performances was significantly better in the simple (64.5%) compared to the complex (56.5%) (*F*(1,7) = 6.2, *p* = 0.042) condition. There was no main effect of mode (*F*(1,7) = 0.0, *p* = 0.836) nor was there a reliable interaction between mode and rhythm (*F*(1,7) = 0.3, *p* = 0.778).

#### Influence

**Fig. 8:**
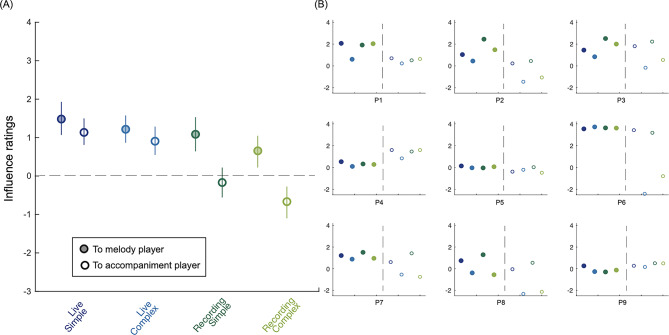
Subjective ratings of perceived influence. (**A**) Participant’s mean influence ratings for the melody player (in filled dots) and for the accompaniment player (in empty dots) where zero indicates neutral influence, -3 indicated ‘negative distraction’ and + 3 ‘positive influence’. Error bars are 1 SD. (**B**) Each individual’s influence ratings to the melody player (filled dots; left panel) and to the accompaniment player (empty dots; right panel).

The overall rating of the influence was 0.775, where the ratings range was from − 3 to 3. Three-way repeated measure ANOVA revealed reliable main effects of rhythm (*F*(1,7) = 6.0, *p* = 0.044), mode (*F*(1,7) = 13.9, *p* = 0.007), and player (*F*(1,7) = 14.6, *p* = 0.007). with no interactions. Inspection of Fig. 8A shows more positive influence in simple meter, and live mode, and with respect to the melody player. This pattern is seen in five of the individual plots (Fig. 8B).

## Discussion

The maintenance of musical ensemble can be accounted for in terms of a first-order linear phase correction model, in which each player proportionally corrects to their partners based on the asynchrony between their most recent notes and the most recent note of each of the other players^1^. In the model, the correction gains used by the players might be subject to a range of different variables. In the present study, we examined the sensitivity of the correction gains of the violinist participant to rhythm (simple vs. complex condition) and mode (live vs. recorded condition).

Individual participants played first violin part with a pre-formed violin duo playing first (melody) and second (accompaniment) violin parts of an excerpt from Haydn Op.74 under the various conditions of interest. The following summarises some key characteristics of how they played. First, participants played slightly ahead of the duo rather than following them. This may be seen as parallelling the sensorimotor synchronisation literature in which finger tapping responses are placed on average tens of milliseconds ahead of a metronome pulse (subtracting the time of the metronome pulse from the time of the tap results in a negative mean asynchrony NMA^9)^. Few studies have reported mean asynchrony in music ensembles^31^, but it is interesting to note that Rasch^14^ reported a small (5–10 ms) negative mean asynchrony for the violin relative to viola and cello in the two string trios he studied.

In the present study the asynchrony variability was smaller with the duo first violin part (melody) than with the duo second violin (accompaniment). Asynchrony variability was also smaller in the simple meter (first and second violin both playing 2/4) than in the complex condition (first violin playing 2/4 second violin playing 6/8). Interestingly, participants exhibited most asynchrony variability with the accompaniment in the live/complex condition. We suggest this is a result of two interacting factors, in that participants had to maintain synchrony with a live (perhaps less predictable) player while being sensitive to the compound 6/8 meter played by the accompaniment player.

Analyses of the estimated phase correction model parameters provided further information about participants’ performance in the ensemble. No statistically significant difference in the participant’s correction gain was found with respect to the two players in the duo even though, as noted above, asynchrony variability was smaller between the participant and melody. This would suggest that similarities in the score alone did not encourage stronger timing adjustments. However, there was an interaction effect between player and rhythm. It revealed contrasting patterns of gain, with participants showing a reduction in gain from simple to compound meter for the accompaniment player, while their gain for the melody player was not affected by rhythmic complexity. This was particularly evident when melody was played live but we also observed a trend in this direction when the melody was recorded. The effect of rhythmic complexity was expected because participants had to estimate the accompaniment’s next tone onset from an alternation between synchronous and syncopated tones. The detection of any discrepancies between tone onsets of the duo gets harder, not only with the complex condition, but also with the increased variability when the melody played live. The results showed that it was easier to correct to an accompaniment when all players were temporally aligned.

One of the goals of the current study was to test the hypothesis that the violinist participant would increase the strength of their correction gain, when playing with a pre-recorded part since the pre-recorded part cannot correct to the participant. In cooperative timing a working assumption might be that all players employ some phase correction. In that case, players might be expected to attempt greater correction to compensate for a particular player who exhibited smaller correction gain to aim for an overall gain value that sums to one. Findings in the literature supports such hypothesis as an optimal strategy to synchronisation. Perhaps a relevant example is Konvalinka et al.^32^ joint finger tapping study where the pairs were unidirectionally or bidirectionally coupled. They found the worst synchronisation performance when correction was unidirectional and the partner’s timing was unpredictable. They therefore concluded that synchronisation behaviour was driven by a mutual, adaptive process between the pair. Contrary to this view, our results showed no greater correction playing to a recording than to a live partner. One possibility is that participants were unsure about the subtle cues marking differences between a recording and a live performance. Our perceptual discrimination results suggest that participants sometimes failed to tell live and recordings apart. Such an explanation would imply that compensatory increase in correction gain to support unidirectional correction is only possible if there is conscious awareness of information flows and correction links in the group.

The individual differences in patterns of gain suggest slight differences in synchronisation strategy but all participants managed to achieve synchronisation with the duo, nevertheless. For example, participants 1, 4, 7, and 8 assigned stronger correction to the live melody player with a compound meter accompaniment but did not use this approach when the melody was recorded. The gains with respect to melody and accompaniment player were negatively correlated during live performances. The strategy here appeared to be choosing to correct to the live melody player that is easier to hear (e.g. melody pitch was higher than the accompaniment tune, melody shared the part with participants, melody player also led the accompaniment player), and in addition, the live player may react to the participant, which may further encourage correction to the melody. Once the reciprocal correction link was removed (i.e. melody recorded), these four participants showed relatively less correction to the melody with a compound meter accompaniment. An alternative strategy, displayed by the gain patterns from the rest of the participants, was to increase correction gain for the compound meter accompaniment especially when both players from the duo were recorded – a strategy that resembled what we originally expected to observe. For the overall stability of the ensemble, participants assigned more weight to synchronise to the compound meter, in order to compensate for the lack of correction from the recordings. We suggest two factors that drive these differences in synchronisation strategy. The first factor, relates to how well a violinist can play (including expertise and familiarity with this particular except in the study), and the second, to their subjective experience performing with the duo, including their sensitivity to picking up discrepancies between a live performance and a recording.

Next, we turn to discuss the average gain parameter estimated for all players, including our participants and the duo who performed in this study, that were generally smaller than the optimal value predicted by the linear phase correction model (i.e. 0.5 when melody was live or 1 when the duo was recorded). Wing et al.^1^ reported under-correction behaviour in that gains were 20% less than the optimal value of 0.25 for a quartet predicted by the linear phase correction model. They suggested this might have arisen because there was appreciable motor variance and this was ignored in their estimation of correction gain. They also noted that as motor variance increases, the optimal value for the correction gain reduces. In the present study, the motor variance attained appreciable levels, exceeding half the value of the timekeeper variance (see Supplementary 1). This may have contributed to participants’ adopting smaller correction gains since motor variance jitters the timing of successive notes (causing asynchrony variance) but, unlike timekeeper variance, does not affect the phase of the following notes.

In contrast to the two professional quartets in Wing et al.^1^, our amateur musicians in this study may not have been as sensitive to temporal asynchronies, were not familiar with their ensemble ‘partners’, and were not instructed to play expressively. In addition, the present study had a particular emphasis on auditory timing for research purposes. Since both conditions (recording and live) relied solely on auditory information, and participants are generally unaccustomed to synchronizing with a recording without visual interaction, differences between live and recorded performance may have been reduced. However, multisensory cues (which were absent in this study) have been shown to improve the quality of coordination between musicians (e.g. with body sway, head position, bowing movements^33–36^, visual cues for a realistic performance setting would be a useful area for future investigation with amateur musicians and players that had not previously played together.

Participants’ subjective reports of perceived influence suggest that players favoured the simple, live source of auditory cues. We showed that participants perceived more positive influence from the melody player, from a simple meter, and from a live player. Although some ratings were neutral (e.g. participant 5 and 9), results generally reflected a negative influence from the compound meter, particularly when the duo was recorded. These results suggest not only that participants preferred predictable inputs, but that their experiences of performing together were also facilitated by mutual correction to some extent. Findings here complemented the behavioural results in that both highlight the positive impact of temporal alignment across players and the importance of a mutual and reciprocated interpersonal relationship during an ensemble performance.

While the present study provides valuable insights into how musicians synchronise, several limitations in the study design should be acknowledged. First, participants were informed at the outset that the melody player could be performing live or could be a recording. This information may have biased their attention toward the first violin part and away from the accompaniment, as reflected in the observed results. By telling participants about potential differences between live and recorded performances of the melody player, they may have consciously adjusted their coordination strategies. Although it might seem logical that they would focus primarily on the changing (melody) part, successful identification of live versus recorded melody performance would also require attention to the temporal relationship between melody and accompaniment. We acknowledge that the information given to the participant places limitations on the strength and generality of conclusions that can be drawn from the findings of the present study. Future research should withhold such information and instead ask participants to rate the ease of coordination with the unison melody, allowing for statistical comparison between live and recorded conditions without introducing expectancy effects. Future studies could also manipulate the parts played by the duo, avoiding the participant player and the melody player playing in unison.

A second limitation in the present study was that the performers could not see each other during the experiment, meaning the setup was not entirely comparable to a typical ensemble performance, which normally involves both visual (e.g. motion) and other auditory interaction (e.g. sniffing to initiate start) cues. However, this design choice was necessary as visual cues would be likely to reveal the conditions to the participants. Moreover, it might be noted that the conditions mimic the conditions of a training tool such as Music minus One^37^ in which a musician rehearses alone while playing along with pre-recorded ensemble parts. In this case, performers rely solely on auditory timing cues without visual information or a click track, making this setup relevant for understanding synchronisation processes in individual practice environments. Future studies could extend this approach by systematically comparing auditory-only conditions with those including visual or gestural information to better capture the full range of real-world ensemble coordination (as is the case, for example, with the PartPlay rehearsal tool^38)^.

A third limitation is that the study focused exclusively on amateur musicians playing classical western style music. Previously, we have studied ensemble performance of professional musicians playing this genre of music in terms of the linear phase correction model. This, for example, revealed a contrast in ensemble style with different roles for the first violin in different quartets^1^. The greater degree of expertise in professional musicians compared to amateurs might be expected to reduce asynchrony variability and increase sensitivity to effects of the different conditions in the present study. However, it also possible that greater levels of expertise would reduce the impact of the accompanist in the complex condition. It might possibly also lead to reduced levels of correction (greater tolerance of deviations from asynchrony). It would be interesting to compare amateur and professional performers to investigate how expertise, experience, and perceptual sensitivity shape synchronization and role adaptation in ensemble contexts. It would also be interesting to evaluate different styles of playing such as more emphasis on expressive interpretative departures from the ensemble. This might even extend to an exploration, perhaps with a different musical excerpt, or the introduction of a swing feel with deviations from the beat as in jazz.

A final limitation is that the analysis approach taken was based on an event-based^39^ view with between-player interactions based on pair-wise linear phase correction to maintain ensemble. In future research on music ensemble, a dynamical approach^40^ could offer an interesting alternative view although not without its critics^41^. A dynamical approach would encourage a view of interactions between players as being essentially continuous in time. Such an approach would also be concerned with scalability of the accounts of the interaction to larger numbers of players where pairwise interactions no longer seem plausible for maintenance of ensemble. For example, a chamber orchestra with 20–30 players, but still without a conductor, might be treated in terms of principles of group dynamics with players treated as unstable oscillators driven by coupling functions, based on both auditory and visual channels. These would bring instrumental sections or subgroups into stable patterns, with sectional interactions proceeding at a higher level, perhaps with additional coupling functions applied in distributed manner across section leaders^42^.

## Conclusion

Adaptive behaviour in a small ensemble is not only initiated by the assignment of musical roles, e.g. with a leader-follower relationship. Rather, we showed that timing correction is a fundamental feature in ensemble performances that occurs automatically. Temporal cues that indicate reciprocated adjustments are non-verbal and subtle; however, our study demonstrated that interpersonal behaviour and perception can be manipulated by similarity in melody, rhythmic complexity, and even constraints on interpersonal timing information flow. In future research it will be interesting to extend the protocol used in the present study and assign participants to also play the accompaniment part to avoid confounding the role of melody and similarity in music. A further interesting direction for research would be to ask about visual communication strategies and methods between players to support their detailed timing as analysed in this paper. This might include, for example, the player’s body sway or other bodily movements such as the upper limbs or head that may contribute to synchronisation timing. In the long term, future research could explore interpersonal synchronisation in larger musical ensembles to examine how coordination strategies might differ from those observed in smaller groups.

## Supplementary Information

Below is the link to the electronic supplementary material.

Supplementary Material 1

## Data Availability

The datasets generated and analysed during the current study are available in the OSF repository, https://osf.io/7gwkh/?view_only=47c930afe3254a3cacd8cadc8df27719.
