# Supplementary material for: Phase correction in violin ensemble performance
Source: Sci Rep. 2026 May 4;16:24231. doi: 10.1038/s41598-026-46205-6 (PMC13443922; doi:10.1038/s41598-026-46205-6)
Supplement: Supplementary file 1 [file 41598_2026_46205_MOESM1_ESM.docx]

**Supplementary Materials**

**Figure 1. Estimation of variance parameters.** Participant player’s estimated motor variance represented by circles, and estimated time-keeper variance represented by stars. Data points are individual participants
